# Adjusting tidal volume to stress index in an open lung condition optimizes ventilation and prevents overdistension in an experimental model of lung injury and reduced chest wall compliance

**DOI:** 10.1186/s13054-014-0726-3

**Published:** 2015-01-13

**Authors:** Carlos Ferrando, Fernando Suárez-Sipmann, Andrea Gutierrez, Gerardo Tusman, Jose Carbonell, Marisa García, Laura Piqueras, Desamparados Compañ, Susanie Flores, Marina Soro, Alicia Llombart, Francisco Javier Belda

**Affiliations:** Anesthesiology and Critical Care Department, Hospital Clínico Universitario of Valencia, Av. Blasco Ibañez, 17, Valencia, CP: 46010 Spain; Section of Anesthesiology and Critical Care, Uppsala University Hospital Uppsala, Uppsala, Sweden; CIBER de Enfermedades Respiratorias, Instituto de Salud Carlos III, Madrid, Spain; Department of Anesthesiology, Hospital Privado de Comunidad, Mar de Plata, Argentina; Clinical Research Foundation, Hospital Clínico Universitario of Valencia, Valencia, Spain; Pathological Anatomy Department, Hospital Clínico Universitario of Valencia, Valencia, Spain; Radiology Department, Hospital Clinico Universitario of Valencia, Valencia, Spain; Clinical Research Foundation, Hospital Clínico Universitario of Valencia, Valencia, Spain

## Abstract

**Introduction:**

The stress index (SI), a parameter derived from the shape of the pressure-time curve, can identify injurious mechanical ventilation. We tested the hypothesis that adjusting tidal volume (VT) to a non-injurious SI in an open lung condition avoids hypoventilation while preventing overdistension in an experimental model of combined lung injury and low chest-wall compliance (Ccw).

**Methods:**

Lung injury was induced by repeated lung lavages using warm saline solution, and Ccw was reduced by controlled intra-abdominal air-insufflation in 22 anesthetized, paralyzed and mechanically ventilated pigs. After injury animals were recruited and submitted to a positive end-expiratory pressure (PEEP) titration trial to find the PEEP level resulting in maximum compliance. During a subsequent four hours of mechanical ventilation, VT was adjusted to keep a plateau pressure (Pplat) of 30 cmH2O (Pplat-group, n = 11) or to a SI between 0.95 and 1.05 (SI-group, n = 11). Respiratory rate was adjusted to maintain a ‘normal’ PaCO2 (35 to 65 mmHg). SI, lung mechanics, arterial-blood gases haemodynamics pro-inflammatory cytokines and histopathology were analyzed. In addition Computed Tomography (CT) data were acquired at end expiration and end inspiration in six animals.

**Results:**

PaCO2 was significantly higher in the Pplat-group (82 versus 53 mmHg, *P* = 0.01), with a resulting lower pH (7.19 versus 7.34, *P* = 0.01). We observed significant differences in VT (7.3 versus 5.4 mlKg^−1^, *P* = 0.002) and Pplat values (30 versus 35 cmH2O, *P* = 0.001) between the Pplat-group and SI-group respectively. SI (1.03 versus 0.99, *P* = 0.42) and end-inspiratory transpulmonary pressure (P_TP_) (17 versus 18 cmH2O, *P* = 0.42) were similar in the Pplat- and SI-groups respectively, without differences in overinflated lung areas at end- inspiration in both groups. Cytokines and histopathology showed no differences.

**Conclusions:**

Setting tidal volume to a non-injurious stress index in an open lung condition improves alveolar ventilation and prevents overdistension without increasing lung injury. This is in comparison with limited Pplat protective ventilation in a model of lung injury with low chest-wall compliance.

**Electronic supplementary material:**

The online version of this article (doi:10.1186/s13054-014-0726-3) contains supplementary material, which is available to authorized users.

## Introduction

Lung protective ventilation limiting airway plateau pressure (Pplat) ≤30 cmH_2_O minimizes alveolar overdistension and reduces mortality in acute respiratory distress syndrome (ARDS) patients [1,2]. However, alveolar overdistension is more directly dependent on transpulmonary pressure (P_TP_), that is, the distending force in the lung. This means that the same Pplat can result in substantially lower P_TP_ in conditions of increased pleural pressure such as ARDS patients with reduced chest wall compliance (Ccw) or intra-abdominal hypertension (IAH). In those conditions several methods to individualize open-lung positive end-expiratory pressure (OL-PEEP) have been proposed to optimize lung mechanics and to improve gas exchange while reducing lung injury [3-5]. However, once OL-PEEP is adjusted, limiting Pplat may be challenging and often requires a tidal volume (VT) restriction that may induce hypoventilation and respiratory acidosis and on the other hand Pplat >30 cmH_2_O theoretically produces overdistension and lung injury. Apart from limiting Pplat, there is no validated simple clinical tool for detecting lung overdistension at the bedside. Hence, there is need for alternative methods for individualizing VT. The stress index (SI), assessed during constant inspiratory flow, analyzes the shape of the pressure-time curve. It can indicate tidal overdistension when displaying an upward concavity, tidal recruitment when displaying a downward concavity and non-injurious ventilation when the shape follows a straight line [6]. Several experimental and clinical studies have shown that when ventilatory parameters are adjusted to a non-injurious SI (0.95 to 1.05) [7] there is a decrease in lung inflammation and lung injury [6-8]. Until now the SI has been mainly evaluated to guide either PEEP alone or VT and PEEP simultaneously but not in conditions of reduced Ccw [6,8,9].

Furthermore, it is unclear whether SI can be useful for guiding VT selection together with OL-PEEP during lung protective ventilation as it has never been evaluated in this context. This could be helpful in clinical practice as OL-PEEP levels are generally higher, especially in the context of reduced chest wall compliance [10]. In this condition even protective tidal volumes may result in higher than recommended plateau pressures whereas limiting VT to a protective plateau pressure may result in hypoventilation. We hypothesized that individualizing VT to a non-injurious SI could be useful to avoid hypoventilation while preventing overdistension despite a Pplat >30 cmH_2_O. To test our hypothesis we compared in an animal model of lung injury and reduced Ccw, in an open lung condition, the individualized VT to a non-injurious SI with the limited Pplat protective ventilation.

## Materials and methods

This experimental study was approved by the Ethics Committee of Animal Experimentation of the Valencia University, Valencia, Spain (trial registration code A13291133306734). We studied 22 Landrace/Large white crossbred pigs weighing 30 to 40 kg.

An additional file shows the experimental protocol [see Additional file 1].

### Anesthesia management

Animals were premedicated with ketamine (15 mg), medetomidine (2 mg), and azaperone (2 mg). Anesthesia was induced by midazolam (20 mg) and fentanyl (0.03 mg kg^−1^) and maintained with midazolam (3 mg kg^−1^ min^−1^), remifentanil (0.15 μg kg^−1^ min^−1^) and cisatracurium (0.08 mg kg^−1^ h^−1^). Mechanical ventilation using a Servo-i ventilator (Maquet Critical Care AB, Solna, Sweden) was delivered with a cuffed tube in the supine position in a volume-controlled (VCV) mode with constant inspiration flow (square wave), expiratory VT of 8 ml kg^−1^, PEEP 5 cmH_2_O inspiratory/expiratory ratio 1:2 with an inspiratory pause of 10%, FIO_2_1, and respiratory rate (RR) adjusted to a PaCO_2_ of 35 to 65 mmHg. Body temperature was maintained at 36°C to 37°C with heated blankets.

### Instrumentation

A 3-Fr thermodilution catheter (PV2013L07-A, Pulsion Medical Systems AG, Munich, Germany) was placed in the right femoral artery for cardiac output monitoring. A 7-Fr double-lumen catheter (AK-22702-P1A, ARROW International, Inc., Morrisville, NC, USA) was inserted into the right or left internal jugular vein for drugs and fluid infusion and transpulmonary thermodilution (TPTD).

### Experimental model

Lung injury was induced through repeated lung lavages with 30 ml kg^−1^ of warm (37°C) normal saline while the animals were maintained in a supine position. Lavages were repeated until a PaO_2_/FIO_2_ ratio of <200 mmHg was reached. As previous studies have demonstrated, this model of lung injury promotes lung collapse in gravity-dependent regions [11] but has little effect on lung permeability or inflammation [12-14], although cytokines are usually detected in the lavage fluid.

To establish the IAH, a midline mini-laparotomy was performed to introduce a trocar (Auto Suture™ Blunt Tip trocar 10mmm, Covidien, Mansfield, MA, USA) for air insufflation [15]. The intra-abdominal pressure (IAP) was measured continuously with a pressure transducer calibrated to atmospheric pressure measured at the mid-thoracic level. The IAP was maintained between 25 and 27 mmHg [16].

### Respiratory monitoring

Respiratory parameters, expiratory VT and minute ventilation (VE), RR, airway pressures (Paw), Pplat, P_TP_, pleural pressure, PEEP and SI were obtained from a FluxMed monitor (MBMed, Buenos Aires, Argentina) that includes a pneumotachograph placed between the endotracheal tube and the ‘Y’ piece of the breathing circuit. End-inspiratory and end-expiratory pressures were obtained after a pause of three seconds.

Esophageal pressure was measured by an esophageal catheter (MBMed) inserted following the manufacturer’s recommendations. The catheter’s optimal position was confirmed by a positive occlusion test [17]. During measurements, the esophageal balloon was inflated with 1 ml of air. P_TP_ was calculated using the standard formula as: P_TP_ = Pplat - pleural pressure. Static respiratory system compliance was calculated as Crs = VT/(Pplat – PEEP). Lung compliance was calculated as C_L_ = VT/(end-inspiratory P_TP_ – end-expiratory P_TP_), and Ccw was calculated as VT (end-inspiratory pleural pressure – end-expiratory pleural pressure). The presence of auto-PEEP was evaluated in real-time by observing the flow-volume curves on the FluxMed monitor [18]. In the presence of an interrupted expiratory flow, that is, when inspiratory flow began before expiratory flow ceased (that is, reached zero), auto PEEP was assumed to be present.

SI was measured every 30 seconds during the study period as previously described [8]. During the constant flow portion, the inspiratory pressure-time relation can be described by a power equation:

$$ {\mathrm{P}}_{\mathrm{TP}} = \mathrm{a}\ \mathrm{x}\ {\mathrm{t}}^{\mathrm{b}} + \mathrm{c} $$

where the coefficient a represents the slope of the pressure-time relationship in the time 0 to time 1 interval, and the coefficient c is the value of pressure at time 0. The coefficient b (SI) is a dimensionless number that describes the shape of the pressure-time curve.

A pressure-time curve displaying an upward concavity (SI >1.05) indicates tidal overdistension, a downward concavity (SI <0.95) tidal recruitment, whereas a straight line (0.95 > SI <1.05) is indicative of less injurious ventilation.

Arterial blood gases were taken at each measurement time-point (i-STAT Analyzer, Abbott Laboratories, East Windsor, NJ, USA).

### Hemodynamic monitoring and management

A PiCCO monitor (Pulsion Medical Systems AG) was used for hemodynamic monitoring. The cardiac index (CI) was obtained by triple TPTD using 10 ml of cold saline, which also provided the following derived parameters: intra-thoracic blood volume index (ITBVI) extravascular lung water index (EVLWI) and pulmonary vascular permeability index (PVPI). The mean arterial pressure (MAP) and heart rate (HR) were recorded continuously.

Hemodynamic stability was maintained following a standard protocol as previously described [19] prior to the start of the experimentation.

Throughout the study, animals received a continuous crystalloid (4 to 6 ml kg^−1^ h^−1^ Ringer’s-Lactate solution) infusion.

### Experimental protocol

The experimental protocol is described in Additional file 1. The experimental protocol lasted a total of 340 minutes. Ventilatory parameters were adjusted according to the described baseline ventilation but limiting RR to a maximum of 35 bpm again assuring a PaCO_2_ between 35 and 65 mmHg. After thirty minutes stabilization after inducing lung injury and IAH, the experimental protocol was performed as follows.

### PEEP adjustment: recruitment maneuver and PEEP titration

The recruitment maneuver (RM) was performed as follows. The ventilator was switched to pressure-control ventilation (PCV) with a driving pressure of 20 cmH_2_O, a PEEP of 5 cmH_2_O and 10 bpm. PEEP was then increased in 5-cmH_2_O steps, each lasting 10 breaths, until reaching an inspiratory opening pressure of 50 cmH_2_O (that is, 20 cmH_2_O of driving pressure and 30 cmH_2_O of PEEP). This opening pressure was then maintained for 20 breaths (that is, two minutes) [20,21]. The RM was immediately followed by a decremental PEEP trial for PEEP titration. The ventilator was switched to VCV with 6 ml kg^−1^, a RR of 20 bpm and a PEEP of 30 cmH_2_O. PEEP was decreased in 2-cmH_2_O steps, each maintained for two minutes, until the best Crs was detected. Thereafter, a new RM was performed as described above to re-open alveoli collapsed during the decremental PEEP titration. The ventilator was switched back to VCV, and the open-lung PEEP level (PEEP with best Crs + 2 cmH_2_0) [21] was established and maintained during the rest of the experimental period. Ventilation according to each randomly assigned group then proceeded for a four-hour period and a full set of measurements was taken at the beginning (baseline, T0), and at 60 (T1), 120 (T2), 180 (T3) and 240 (T4) minutes. [see Additional file 1].

### Tidal volume adjustment

VT adjustment was initiated one minute after open lung PEEP. For the adjustment of tidal volume, animals were then randomly assigned to a Pplat-group or a SI-group.

In the Pplat-group, VT was adjusted to obtain a Pplat = 30 cmH_2_O. The VT was readjusted in steps of 1 ml kg^−1^ every five minutes during the experiment if needed to maintain the target Pplat.

In the SI-group, VT was adjusted to maintain the SI between 0.95 and 1.05. The VT was readjusted in steps of 1 ml kg^−1^ every five minutes during the experiment if needed to maintain the target SI.

VT and RR were not modified for pH management unless pH ≤7.15. When pH was ≤7.15 and RR <35, RR was increased in steps of 1 bpm to maintain a pH >7.15. When pH was ≤7.15 and RR = 35 rpm, VT was increased in steps of 1 ml kg^−1^ irrespective of Pplat or SI.

### Computed tomography scanning and analysis

At the end of the experimental protocol six animals (three of the Pplat-group and three of the SI-group) were transferred to the computed tomography (CT) scanner without interrupting ventilation. Spiral CT scans (120 kV, 110 mA) of the total lungs were performed during end-expiration and end-inspiration holds using a Discovery CT750 HD (GE Healthcare). Images were reconstructed in 3-mm slices using a standard filter for lung parenchyma for the CT-image edition. Four sections of the thorax to be imaged were selected on the scout view: level 1: aortic arch, level 2: heart, level 3: main bronchi and level 4: just above the diaphragm. Each level was divided in two regions of interest (ROI): V: ventral and D: dorsal. Lung aeration was assessed by a radiologist who was blinded to group identity by measuring normally aerated, poorly aerated, nonaerated, and overinflated lung volumes as previously described [22]. Nonaerated lung was defined by lung densities ranging between -100 and +100 Hounsfield Units (HU), poorly aerated lung by lung densities ranging between -100 and -500 HU, normally aerated lung by lung densities ranging between -500 and -900 HU and overinflated lung by lung densities ranging between -900 and -1000 HU. The amount of the different densities was expressed as a percentage of the total lung parenchyma in the region analyzed. The threshold of a clinical significant percent of a lung density compartment over the total lung parenchyma in the region analyzed was 10% [21].

### Inflammation and histopathology

An additional file shows the description of Material and methods for the determination of inflammatory markers and histopathology [see Additional file 2].

### Statistical analysis

Data were entered into the statistical package SPSS version 15.0 (IBM, Chicago, IL, USA). The Kolmogorov-Smirnov and Levene’s test were used to determine normality and homogeneity, respectively. When the homogeneity hypothesis was rejected (test *P*-value <0.05), the Mann Whitney U test and a Friedman test were applied. If not rejected, a Student’s t-test and analysis of variance (ANOVA) were performed. For multiple comparisons, the Bonferroni correction was used to fit a type I risk to the chosen significance level (α = 5%). Data are presented as the mean (standard deviation, SD) if normally distributed and as median (interquartile range, IQR) otherwise.

## Results

The mean (SD) animal weight was 32 (2) kg for the SI-group and 31 (2) kg for the Pplat-group. The open-lung PEEP level was found to be 17 (2) cmH_2_O for the SI-group and to be 18 (2) cmH_2_O for the Pplat-group (*P* = 0.69). Temperature was maintained between 36°C and 37°C in all animals.

The animal model of lung injury and IAH as previously described [10,21] reduced Crs by 33% (*P* <0.001; 95% confidence interval (CI) 2 to 11), Ccw by 57% (*P* <0.001; 95% CI 41 to 49) and C_L_ by 62% (*P* <0.001; 95% CI 10 to 18). Also, Pplat was increased by 56% (*P* <0.001; 95% CI 9 to 17) together with an increase of 40% of the P_TPEI_ (*P* <0.001; 95% CI 6 to 11) and 47% of the EVLWI (*P* <0.001; 95% CI 3 to 14). The oxygenation decreased by 66% (*P* <0.001; 95% CI 235 to 422). The changes in the respiratory mechanics, oxygenation and extravascular lung water produced by the saline lavage and air-insufflation remained constant during the experimentation ensuring a stable experimental model.

### Effects of ventilatory strategy on gas exchange and ventilatory mechanics

**Figure 1:**
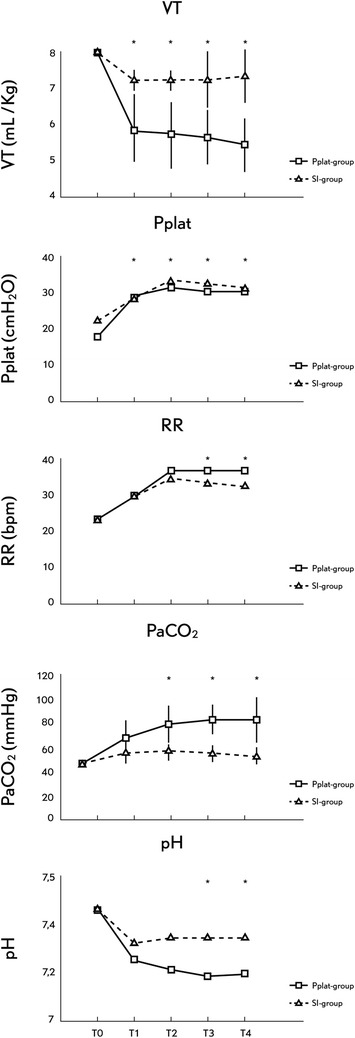
**Changes in the principal variables during the protocol.** T_0_: Baseline . T_1_: 60, T_2_: 120, T_3_: 180 and T_4_: 240 minutes after protocol defined ventilation was started. Data are presented as mean (SD). VT: tidal volume, Pplat: plateau pressure, RR: respiratory rate, PaCO_2_: arterial partial pressure of CO_2_, pH: acid-base state. *When significant difference (*P* <0.05) between Pplat-group versus SI-group.

PaCO_2_ was significantly higher in the Pplat-group resulting in a lower pH (Table 1). In the Plat-group, the PaCO_2_ remained >65 mmHg during the entire experimental period despite reaching the maximum per-protocol allowed RR since T1. In the SI-group, PaCO_2_ was <65 mmHg and pH remained >7.15 in all animals during the experimentation. In the SI-group, the RR was readjusted every hour after arterial blood gases but maximum RR allowed was not reached. In two animals of the Pplat-group, VT had to be increased starting at T2 to try to reach the target pH >7.15 resulting in a Pplat >30 cmH_2_0, but in only one animal was SI >1.05, suggesting tidal overdistension. In all the SI-group animals, Pplat was >30 cmH_2_O. Oxygenation was similar in both groups during the experimental period (Table 1, Figure 1).

**Table 1:** **Respiratory parameters**

**Parameters**	**T**_**0**_	**T**_**1**_	**T**_**2**_	**T**_**3**_	**T**_**4**_
VT					
Pplat-group	8 (0)	5.8 (1.1)*	5.7 (0.9)*	5.6 (0.8)*	5.4 (0.8)*
SI-group		7.2 (0.2)	7.2 (0.2)	7.2 (0.8)	7.3(0.7)
*P*-value	8 (0)	0.02	0.04	0.3	0.002
RR					
Pplat-group	22 (2)	32 (3)	35 (0)	35 (0)*	35 (0)*
SI-group	22 (2)	31 (3)	33 (2)	32 (1)	31 (1)
*P*-value	0.79	0.07	0.10	0.04	<0.001
VE					
Pplat-group	5.4 (0.2)	5.8 (0.2)*	5.9 (0.2)*	6.2 (0.2)*	6.4 (0.2)*
SI-group	5.5 (0.2)	6.1 (0.2)	6.8 (0.1)	7.3 (0.3)	7.3 (0.2)
*P*-value	0.48	0.001	0.001	0.001	0.001
SI					
Pplat-group	1.05 (0.08)	0.90 (0.12)	0.95 (0.13)	0.97 (0.13)	1.03 (0.12)
SI-group	1.01 (0.04)	1.01 (0.06)	0.99 (0.06)	0.98 (0.05)	0.99 (0.07)
*P*-value	0.58	0.29	0.66	0.95	0.42
Pplat					
Pplat-group	18 (2)	29 (3)*	31 (2)*	30(1)*	30 (1)*
SI-group	18 (3)	35 (3)	35 (2)	36 (2)	35 (2)
*P*-value	0.91	0.001	0.002	0.001	0.001
P_TPEE_					
Pplat-group	2(1)	5 (3)	6 (3)	6 (3)	5(3)
SI-group	2 (1)	4 (2)	4 (2)	4 (3)	5 (4)
*P*-value	0.81	0.34	0.32	0.45	0.61
P_TP EI_					
Pplat-group	7 (1)	15 (2)	15 (2)	16 (2)	17 (1)
SI-group	6 (2)	17 (3)	17 (3)	18 (2)	18 (3)
*P*-value	0.56	0.49	0.17	0.22	0.42
ΔP_TP_					
Pplat-group	5 (2)	11 (6)	9 (2)	10 (3)	10 (3)
SI-group	5 (2)	13 (2)	12 (4)	14 (4)	13 (4)
*P*-value	0.74	0.81	0.08	0.18	0.40
Crs					
Pplat-group	20 (3)	13 (4)	14 (3)	14 (3)	13 (3)
SI-group	21 (3)	14 (2)	15 (2)	16 (2)	15 (3)
*P*-value	0.72	0.54	0.63	0.31	0.35
Ccw					
Pplat-group	80 (6)	34 (4)	33 (3)	32 (3)	32 (3)
SI-group	80 (5)	34 (3)	33 (5)	33 (4)	34 (4)
*P*-value	0.92	0.89	0.86	0.24	0.19
C_L_					
Pplat-group	46 (3)	18 (1)	18 (4)	18 (5)	15 (3)
SI-group	50 (5)	18 (3)	18 (3)	18 (2)	18(2)
*P*-value	0.51	0.69	0.73	0.59	0.24
pH					
Pplat-group	7.45 (0.0)	7.25 ( 0.0)*	7.21 (0.0)*	7.18 (0.0)*	7.19 (0.0)*
SI-group	7.45 (0.1)	7.32 (0.0)	7.34 (0.1)	7.34 (0.0)	7.34 (0.1)
*P*-value	0.68	0.09	0.004	0.003	0.01
PaO_2_/FiO_2_					
Pplat-group	482 (122)	151 (40)	179 (49)	225 (80)	220 (82)
SI-group	515 (50)	188 (68)	220 (62)	214 (68)	230 (78)
*P*-value	0.58	0.31	0.52	0.81	0.73
PaCO_2_					
Pplat-group	44 (7)	68 (15)	78 (15)*	83 (12)*	82 (19)*
SI-group	47 (8)	54 (7)	56 (6)	54 (7)	53 (7)
*P*-value	0.54	0.12	0.02	0.002	0.01

Time-points T_0_: Baseline measurement, 10 minutes after protocolized baseline ventilatory parameters, before lung injury; T_1_: 60; T_2_: 120; T_3_: 180; and T_4_: 240 minutes after protocolized ventilatory parameters (open lung PEEP and protocolized VT corresponding to the study group) were adjusted. Data are presented as mean (SD). VT: tidal volume (ml/kg); RR: respiratory rate (bpm); VE: minute volume (L); SI: stress index; Pplat: plateau pressure (cmH_2_O); P_TPEE_: end-expiratory transpulmonary pressure (cmH_2_O); P_TPEI_: end-inspiratory transpulmonary pressure (cmH_2_O); ΔP_TP_: delta-transpulmonary pressure (end-inspiratory – end-expiratory) (cmH_2_O); Crs: respiratory system compliance (ml/cmH_2_O); C_L_: lung compliance (ml/cmH_2_O); Ccw: chest wall compliance (ml/cmH_2_O); pH: acid-based state; PaO_2_/FiO_2_: arterial oxygen tension to inspiratory oxygen fraction index (mmHg); PaCO_2_: arterial carbon dioxide tension (mmHg). *When significant difference (*P* <0.05) between Pplat-group versus SI-group.

**Figure 2:**
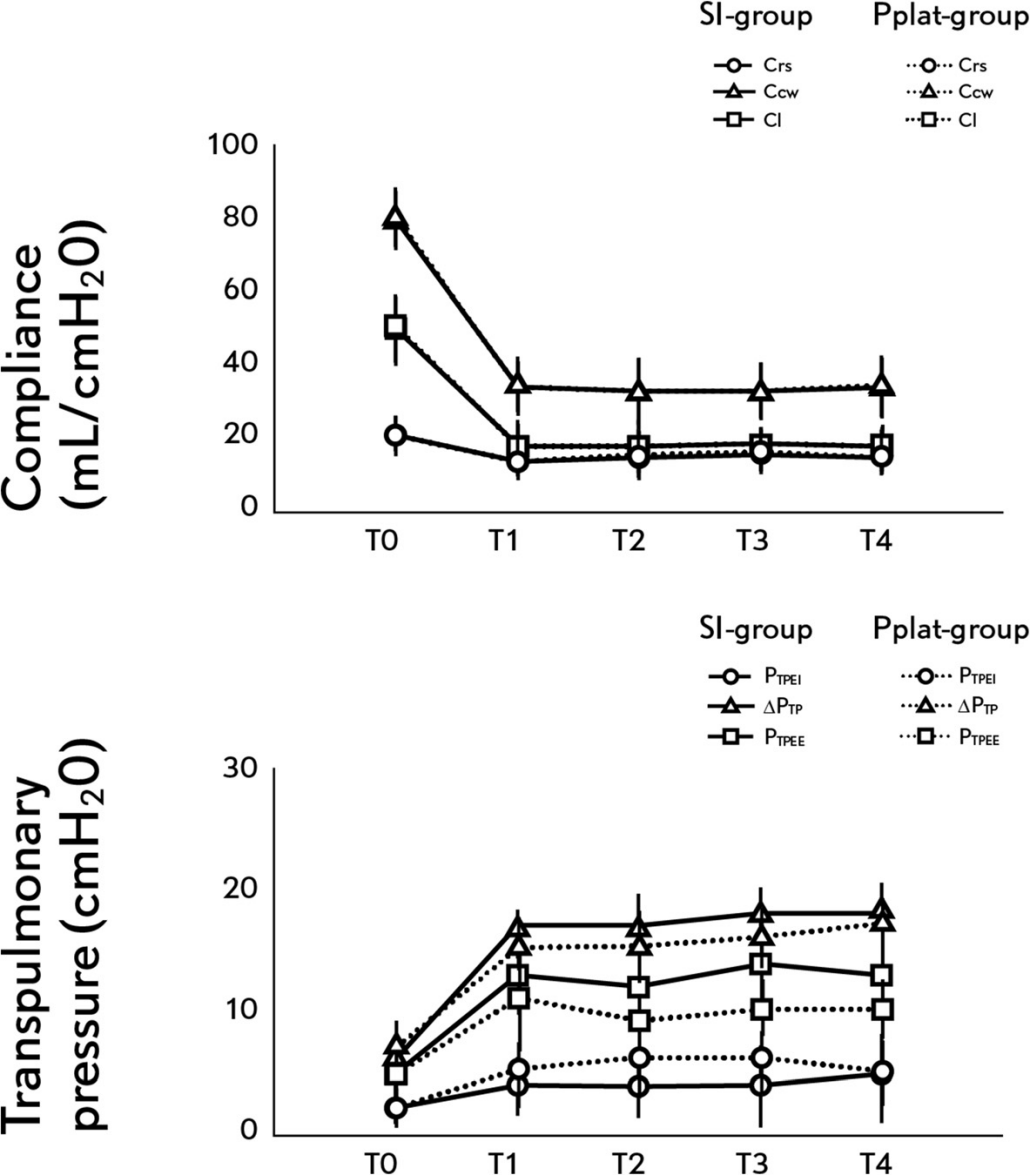
**Changes in lung mechanics during the experimental protocol.** Dashed line: SI-group, pointed line: Pplat-group. Upper panel: compliance. Triangle: chest wall compliance, square: lung compliance, circle: respiratory system compliance. Lower panel: transpulmonary pressure. Triangle: end-inspiratory transpulmonary pressure, square: delta transpulmonary pressure, circle: end-expiratory transpulmonary pressure. T_0_: Baseline. T_1_: 60, T_2_: 120, T_3_: 180 and T_4_: 240 minutes after protocol defined ventilation was started. *P <0.05 Pplat-group versus SI-group.

There was a significant difference in Pplat, VT, VE and RR between both groups at all measurement points (Table 1). While in the Pplat-group the Pplat was controlled with values = 30 cmH_2_O, in the SI-group Pplat was not controlled, with mean values around 35 cmH_2_O during the entire experimental protocol. The SI-group had higher VTs than the Pplat-group. The maximal differences in VT between groups were around 1 to 2 ml kg^−1^. No significant differences between groups were found in the SI, end-expiratory, end-inspiratory and delta P_TP_ and for Crs, Ccw and C_L_ (Table 1, Figure 2). None of the study animals developed auto-PEEP during the study period.

### Effects of ventilatory strategy on lung densities

**Figure 3:**
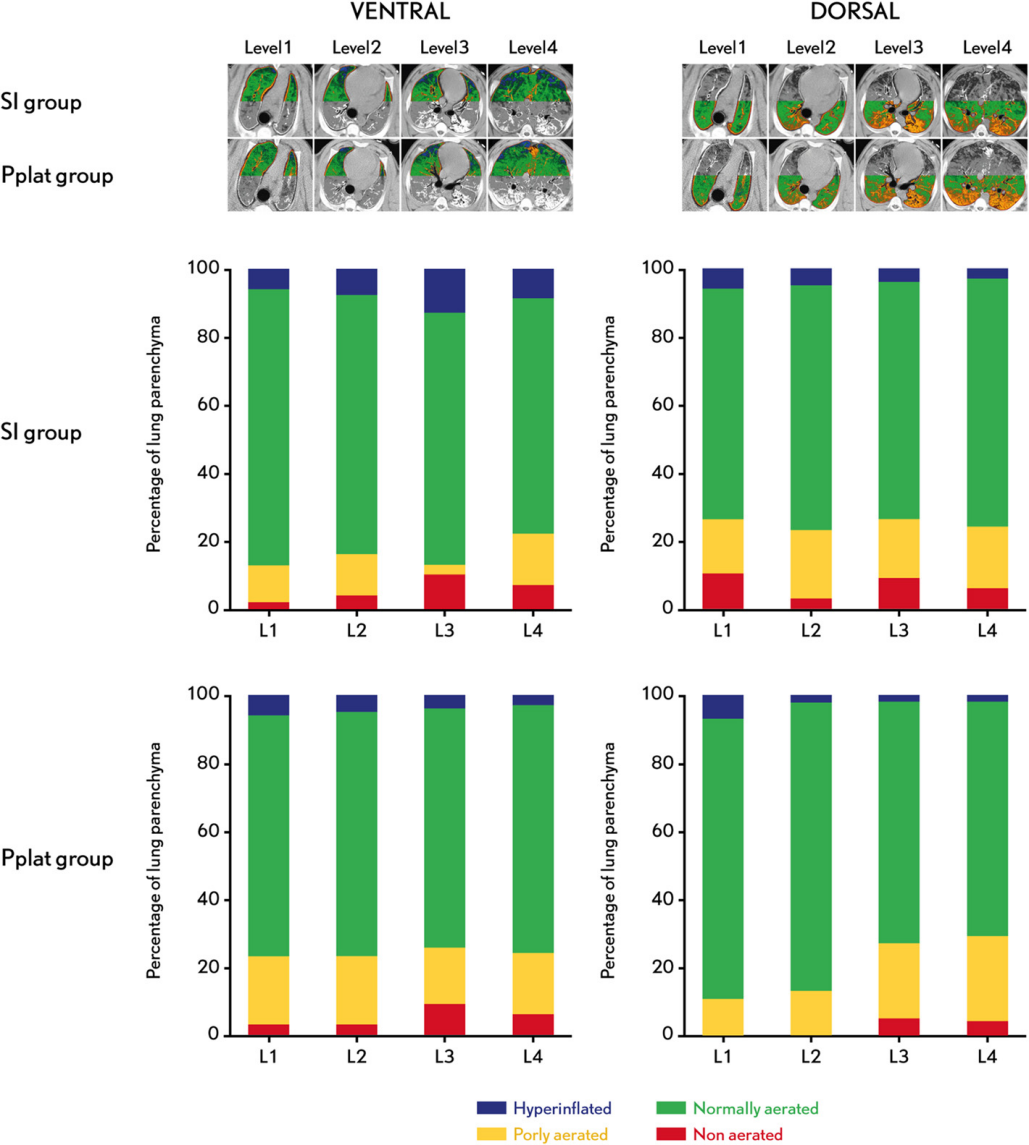
**Computed tomography scan and distribution of the ventilation.** Left side: Ventral ROI, right side: Dorsal ROI. SI-group: VT adjusted to SI between 0.95 and 1.05. Pplat-group: VT adjusted to Pplat = 30 cmH_2_O. Level 1: aortic arch, level 2: heart, level 3: main bronchi and level 4: just above the diaphragm. HU: Hounsfield units. ROIs, regions of interest.

There were no differences in lung aeration between the SI- and Pplat-groups at the four lung levels analyzed. No differences were found between the two ROIs in the four levels analyzed. There were no hyperinflated and non-aerated lung areas in any of the four levels analyzed in the two ROIs, except in the SI-group in level 3 ventral ROIs that presented 12% of non-aerated lung areas (Figure 3).

### Effects of ventilatory strategy on hemodynamics

Animals remained hemodynamically stable. The CI, ITBVI, PAM and FC did not differ between groups. The alveolar recruitment maneuver and PEEP titration did not produce a CI decrease in any pig (Table 2).

**Table 2:** **Haemodynamic parameters**

**Parameters**	**T**_**0**_	**T**_**1**_	**T**_**2**_	**T**_**3**_	**T**_**4**_
IC					
Pplat-group	3.0 (0.9)	2.6 (0.3)	3.0 (0.3)	2.5 (0.8)	2.9 (0.5)
SI-group	3.2 (0.8)	2.7 (0.4)	2.6 (0.4)	2.4 (0.2)	2.5 (0.4)
*P*-value	0.69	0.76	0.08	0.86	0.16
AM					
Pplat-group	103 (11)	99 (12)	105 (8)	93 (15)	91 (28)
SI-group	101 (7)	97 (9)	97 (17)	103 (19)	101 (18)
*P*-value	0.72	0.81	0.41	0.40	0.56
FC					
Pplat-group	74 (8)	72 (13)	78 (25)	83 (38)	88 (25)
SI-group	85 (15)	64 (7)	70 (28)	74 (28)	82 (35)
*P*-value	0.19	0.24	0.64	0.38	0.77
ITBVI					
Pplat-group	521 (123)	492 (41)	551 (63)	542 (195)	568 (72)
SI-group	528 (105)	580 (100)	676 (107)	660 (153)	644 (107)
*P*-value	0.93	0.10	0.05	0.32	0.07
EVLWI					
Pplat-group	8 (2)	18 (5)	15 (3)	14 (3)	15 (4)
SI-group	8 (1)	17 (2)	18 (4)	18 (4)	18 (4)
*P*-value	0.86	0.78	0.34	0.18	0.27
PVPI					
Pplat-group	1.9 (0.8)	3.8 (1.4)	3.5 (0.8)	2.7 (0.7)	2.9 (0.8)
SI-group	2.2 (0.6)	3.2 (0.6)	3.0 (0.9)	2.8 (0.5)	2.8 (0.5)
*P*-value	0.61	0.44	0.40	0.68	0.93

Time-points T_0_: Baseline measurement, 10 minutes after protocolized baseline ventilatory parameters, before lung injury. T_1_: 60, T_2_: 120, T_3_: 180 and T_4_: 240 minutes after protocolized ventilatory parameters (open lung PEEP and protocolized VT corresponding to the study group) were adjusted. Data are presented as mean (SD). IC: cardiac index (ml/min/m^2^), MAP: mean arterial pressure (mmHg), FC: cardiac frequency (bpm), ITBVI: intra-thoracic blood volume indexed to predicted body weight (ml/m^2^), EVLWI: extravascular lung water indexed to predicted body weight (ml/m^2^), PVPI: pulmonary vascular permeability index.

### Effects of ventilatory strategy on inflammatory markers

**Figure 4:**
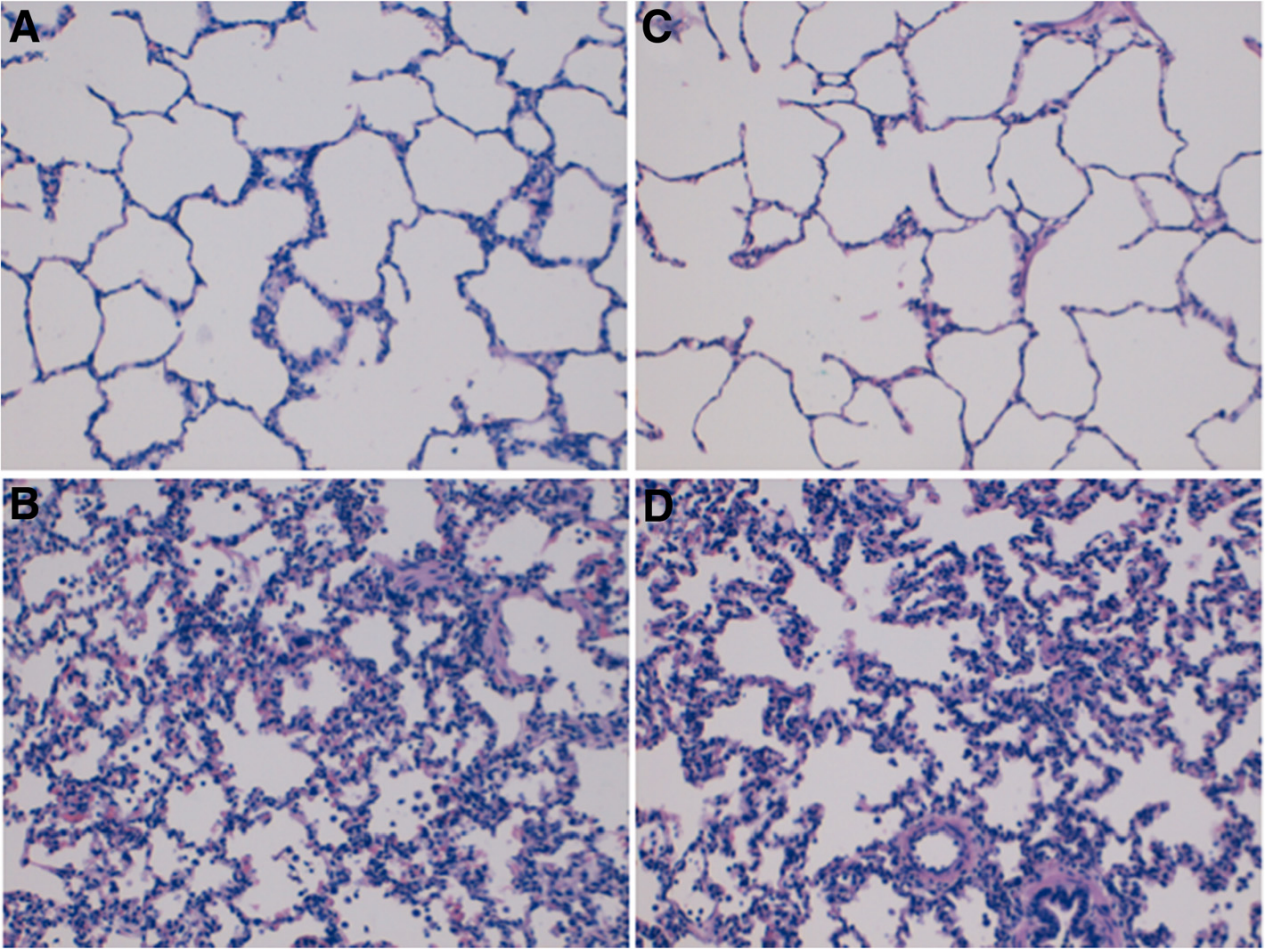
**Lung tissue histopathology. A)** (SI-group) and **C)** (Pplat-group) right ventral sample: mild aggregation of neutrophils and hyaline membrane formation. **B)** (SI-group) and **D)** (Pplat-group) right dorsal sample: severe alveolar congestion hemorrhage, neutrophil airspace infiltration and aggregation and vessel wall infiltration, hyaline membrane formation without differences between groups. [See Additional file 2].

No differences were found in the concentrations of TNF-α and IL-8 between the SI-group and Pplat-group. There were no histopathological differences between the groups. In both groups, the lung damage score was higher in the dorsal lung samples. Neither alveolar wall thickening nor hyaline membrane were observed in any group (Figure 4). An additional file shows the results of broncho-alveolar and plasma cytokines and histopathological analysis [see Additional file 2].

## Discussion

The major finding of our study is that adjusting VT to a targeted non-injurious SI value (0.95 > SI <1.05) in an open lung condition as compared with a VT targeted to Pplat ≤30 cmH_2_O, improves alveolar ventilation while it avoids increasing overdistension in the studied model of lung injury with low chest wall compliance. Furthermore, this targeted VT does not increase the risk of VILI due to overdistension compared to protective ventilation aimed at limiting Pplat. To our knowledge, this is the first study that uses the SI to individually adjust VT in combination with lung recruitment and open lung PEEP. Despite a higher absolute Pplat in the SI-group no significant differences were observed in regional overinflation (on CT), global overdistension (according to the P_TP_), biological and histological markers of lung injury and lung edema between the two groups. In this respect SI appears to be a useful alternative at the bedside for optimizing VT during lung protective ventilation in situations of low chest wall compliance in combination with lung recruitment and OL-PEEP.

A lung protective ventilation strategy that limited VT to maintain a Pplat ≤30 cmH_2_O decreased mortality in ALI/ARDS patients [1,2]. However, Pplat is not representative of alveolar overdistension [23]. The true distending force of the lung is the P_TP_. This is especially important in patients with ARDS with a reduced Ccw [24]. As our results demonstrate, targeting VT to a non-injurious SI does not result in a greater overdistension than the one resulting from targeting VT to a ‘protective’ Pplat ≤30 cmH_2_O [25], while the latter strategy increases the risk of hypoventilation in patients with high pleural pressure.

### Selection of VT

Nowadays, several lung protective ventilation to adjust VT had been proposed to avoid overdistension. The most common are the use of standardized low VT or limiting VT to a maximum Pplat [1] but, as has been previously shown, both are inadequate surrogates for lung atelectrauma and overdistension [23]. This may have important implications in patients with low Ccw, such as patients with IAH [8], obese patients or ARDS patients, where adjusting appropriate lung protective ventilation settings can be a difficult clinical challenge [24] and these strategies may often result in excessive hypercapnia and respiratory acidosis.

It has recently been suggested that limiting end-inspiratory transpulmonary pressure to 25 cmH_2_O is an alternative for limiting tidal overdistension [26] but further studies in different lung conditions together with imaging techniques are needed to confirm this threshold. Moreover, monitoring Ptp with an esophageal catheter, even despite the promising results in clinical and experimental studies, still has many practical and theoretical limitations in clinical practice that could alter the measured value and would not reflect transpulmonary pressure [27,28]. Given the limitations of the available bedside monitoring tools there is no optimal strategy to guide the setting of the best VT during lung injury in conditions of reduced chest wall compliance. Individualized setting of VT to a non-injurious SI could be an easy and accurate tool to minimize overdistension while reducing hypoventilation.

Some studies suggest that SI has a reduced accuracy in detecting cyclic overdistension and recruitment when non-pulmonary factors affect lung mechanics [29] or in heterogeneous lungs (ARDS) because these phenomena can occur simultaneously during a tidal breath [30]. Nevertheless, unlike all previous studies using SI to optimize ventilation [7-9,30], with the proposed approach of setting optimal PEEP after lung recruitment, tidal recruitment should be minimized as theoretically only a minimal amount of lung collapse is present. This should improve the ability of SI to detect injurious settings causing overdistension as theoretically this will be the predominant mechanism influencing the abnormal shape of the pressure time curve. This is further reinforced by the recent demonstration that an SI >1.05 indicates injurious ventilation very accurately in ARDS patients, better than the value of plateau pressure [7].

### Assessment of hyperinflation by means of computed tomography scan

In order to confirm our hypothesis, CT scans were made in six animals. Thoracic CT allows for accurate measurement of pulmonary volume distribution and, thus, the influence of VT on hyperinflation [22]. Despite the significantly higher VTs and Pplat >30 cmH_2_O in the SI-group, no differences in regional hyperinflation, considered as more than 10% of hyperinflated lung tissue of the total lung, were found between the groups. CT analysis did not detect any hyperinflation in the different lung regions in either of the groups. This is in agreement with previous experimental and clinical studies in injured lungs in which CT analysis confirmed that SI [6,7] and dynamic respiratory mechanics [31] can be used to optimize ventilatory parameters.

### Overdistension assessment with global indices of lung function

Interestingly, despite the setting of higher VTs in the SI-group and a Pplat >30 cmH_2_O, we did not find any differences in P_TPEI_ between the groups, suggesting no differences in overdistension. Although there is no known theoretical safe upper limit for transpulmonary pressure, suggested protective levels are accepted to be <25 cmH_2_O [26]. In none of the studied animals, in either group, was P_TPEI_ close to these levels. The P_TPEI_ levels we obtained were similar to those obtained by Krebs *et al*. [10] in ARDS patients with IAH, with PEEP levels of 15 to 20 cmH_2_O and a VT of 6 ml kg^−1^. Recently, Talmor *et al*. used P_TPEE_ to individualize PEEP in ARDS patients. They demonstrated that even with a VT of 7 ml kg^−1^ P_TPEI_ values were relatively low (averaging 8 cmH_2_O) but with a Pplat in the higher ‘safe’ limit (29 cmH_2_O) independent of whether PEEP was adjusted conventionally or guided by esophageal pressure [26].

### Effects of VT size in the inflammatory response

It has been well established that injurious mechanical ventilation can trigger a local and systemic inflammatory response, a process known as biotrauma [32-34]. The results obtained in this study with no differences in cytokine levels and histopathological analysis suggest that adjusting VT to 0.95 > SI <1.05 in a re-expanded lung does not increase the risk of VILI, despite the higher than recommended resulting tidal volumes and plateau pressures. This is consistent with previous studies where targeting ventilation to non-injurious SI decreased the risk of VILI [6] despite short observation periods [35-37].

### Effects of VT size on lung edema

ARDS is characterized by an increase in pulmonary edema [38,39]. No differences were found during the study period in EVLWI between the SI- and Pplat-groups, further reinforcing the concept that targeting VT to a non-injurious SI is a useful approach for lung protective ventilation [40].

### Selection of PEEP

Different studies have demonstrated that in ARDS, PEEP titration for the best Crs after lung recruitment (that is, OL-PEEP) minimizes recruitment/derecruitment and overdistension [21,30]. The OL-PEEP found in our study is similar to that obtained in previous experimental [41] and clinical [10] studies with high pleural pressure. The presence of less than 10% non-aerated areas in the CT at end-expiration and the slightly positive P_TPEE_ values confirm the adequate PEEP level used in this experimental model [10,26]. Moreover, it has been suggested that the optimal PEEP level in ARDS and IAH patients is the level reached after adjustment to the best Crs after lung recruitment [42] and interchangeable with a PEEP titration to best Ptp [43].

### Limitations

Our study has some limitations. First, we used a pig model as its use is well established in IAH [41] and ALI/ARDS [20]. However, the behavior of the respiratory system may be very different in critically ill patients, especially in ARDS patients with abdominal hypertension and, therefore, our results must be interpreted with caution. Second, the time spent on both ventilation modes was limited to four hours, and the long-term effects of the proposed strategy are, therefore, not known. This short study period together with the fact that we were comparing two lung protective ventilation strategies may have limited the extent of the inflammatory response and the histopathological changes seen in our results. Third, it is difficult to interpret the concentration of cytokines in bronchoalveolar lavage (BAL) specimens because an unknown amount of cytokines always remain within the cells and because the ideal lavage volume for this purpose has not yet been established. Furthermore, in the analysis of the inflammatory response we did not include mRNA expression of cytokines or myeloperoxidase activity which may have been earlier and specific markers to evaluate the inflammatory response, especially due to the short period spent on mechanical ventilation. Fourth, we measured the mean IAP instead of the recommended end-expiratory measurement [16]. This may have underestimated the real levels of IAP by approximately 2 to 3 mmHg [44].

## Conclusions

Setting VT to a targeted SI non-injurious value (0.95 to 1.05) in an open lung condition improves alveolar ventilation without increasing the risk of overdistension compared with protective ventilation aimed at limiting plateau pressure in a model of lung injury with low chest wall compliance. Our findings, if confirmed in lung injury patients with reduced chest wall compliance, could result in a useful alternative approach to optimize lung protection and set an appropriate tidal volume in these otherwise difficult to ventilate patients.

## Key messages

- Alveolar overdistension depends more on transpulmonary pressure (P_TP_), that is, the distending force in the lung as determined by the alveolar minus pleural pressure, than on the absolute Pplat value.
- In patients with low Ccw (high pleural pressure), maintaining a Pplat ≤30 cmH_2_O may be challenging and often requires a VT restriction inducing hypoventilation and respiratory acidosis.
- Currently, there are no validation tools for use at the bedside to assess tidal overdistension.
- Setting VT to a targeted SI non-injurious value (0.95 to 1.05) in an open lung condition improves alveolar ventilation without increasing the risk of overdistension.

## Additional files

Additional file 1:
**Supplementary information of the experimental protocol.**
Additional file 2:
**Supplementary information of materials and methods and results of inflammation (broncho-alveolar and plama cytokines) and histopathological analysis.**

## Abbreviations

ALI: acute lung injury
ARDS: acute respiratory distress syndrome
Ccw: chest wall compliance
CI: cardiac index
Cl: lung compliance
CT: computed tomography
EVLWI: extravascular lung water index
FIO_2_: inspiratory oxygen fraction
HR: heart rate
HU: Hounsfield units
IAH: intra-abdominal hypertension
IAP: intra-abdominal pressure
IL: interleukin
ITBVI: intra-thoracic blood volume index
MAP: mean arterial pressure
OL-PEEP: open-lung PEEP
PaCO_2_: arterial carbon dioxide partial pressure
PaO_2_/FiO_2_: arterial oxygen partial pressure to inspiratory oxygen fraction
Paw: airway pressure
PCV: pressure-control ventilation
PEEP: positive end-expiratory pressure
Pplat: plateau pressure
PTP: transpulmonary pressure
P_TPEE_: end-expiratory transpulmonary pressure
P_TPEI_: end-inspiratory transpulmonary pressure
PVPI: pulmonary vascular permeability index
RM: recruitment maneuver
ROI: region of interest
RR: respiratory rate
SD: standard deviation
SI: stress index
TNF: tumor necrosis factor
TPTD: transpulmonary thermodilution
VCV: volume control ventilation
VE: minute ventilation
VILI: ventilator-induced lung injury
VT: tidal volume
