# Supplementary figures and images for: Adjusting tidal volume to stress index in an open lung condition optimizes ventilation and prevents overdistension in an experimental model of lung injury and reduced chest wall compliance
Source: Crit Care. 2015 Jan 13;19(1):9. doi: 10.1186/s13054-014-0726-3 (PMC4352239; doi:10.1186/s13054-014-0726-3)

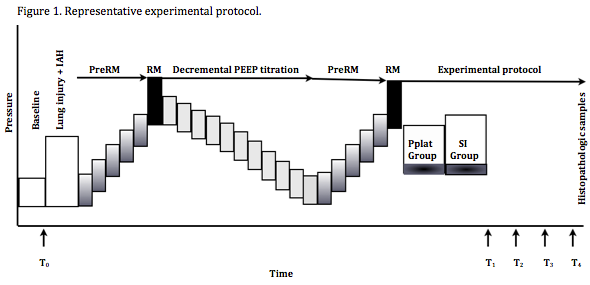

Supplement: Additional file 1: — Supplementary information of the experimental protocol. [file 13054_2014_726_MOESM1_ESM.tiff]
