# Supplementary material for: Adjusting tidal volume to stress index in an open lung condition optimizes ventilation and prevents overdistension in an experimental model of lung injury and reduced chest wall compliance
Source: Crit Care. 2015 Jan 13;19(1):9. doi: 10.1186/s13054-014-0726-3 (PMC4352239; doi:10.1186/s13054-014-0726-3)
Supplement: Additional file 2: — Supplementary information of materials and methods and results of inflammation (broncho-alveolar and plama cytokines) and histopathological analysis. [file 13054_2014_726_MOESM2_ESM.docx]

**Adjusting Tidal Volume to Stress Index in an Open Lung Condition Optimizes Ventilation and Prevents Overdistension in an Experimental Model of Lung Injury and reduced chest wall compliance.**

**Materials and Methods**

*Inflammation and Histopathology*

*Determination of inflammatory marker levels*

Bronchoalveolar lavage (BAL) and blood samples were collected at baseline and at the end of the experimental period. The BAL and blood samples were centrifuged at 3500 rpm at 21°C for 5 min. Following centrifugation, the supernatant was preserved at -80°C for analysis, which was performed in duplicate for all cytokines using Quantikine Enzyme Linked Immunoassay (ELISA) kits (R&D Systems, Minneapolis, USA). The cytokines TNF-α and IL-8 were analyzed.

*Histopathology*

For histopathology, two samples of lung tissue were excised from each animal: right ventral and right dorsal. The samples were fixed in 4% buffered formaldehyde. Subsequently, each sample was divided into three slices and paraffin embedded. Four µm-thick slices were cut, stained with hematoxylin and eosin and analyzed by a pathologist who was blinded to group identity. Ten high power fields (HPFs) were randomly sampled for each slice. The degree of lung damage was assessed using a modified VILI histological scoring system according to the following four items: 1) alveolar congestion, 2) hemorrhage, and 3) infiltration or aggregation of neutrophils in airspace or the vessel wall, and 4) thickness of the alveolar wall/hyaline membrane formation. Each item was graded according to a five-point scale: 0 = minimal damage, 1 = mild damage, 2 = moderate damage; 3 = severe damage; and 4 = maximal damage. The degree of lung damage was assessed by the sum of scores, with a range of 0 to 16. An overall score of lung damage was obtained based on the sum of all scores, and then a median was determined (three slices from each lung sample, two samples for animal and eight animals per group)

**Figure legends.**

**Figure 1.** Experimental protocol. Time-points T_0_: Baseline measurement, 10 minutes after baseline ventilation, before lung injury. T_1_: 60, T_2_: 120, T_3_: 180 and T_4_: 240 min after the specific ventilation protocols in each group were applied. IAH: Intra-abdominal hypertension, RM: Recruitment maneouver.

Table 1. Broncho-alveolar and Plasma cytokines.

|  | **T_0_** | **T_4_** |
| --- | --- | --- |
| TNFalpha (Plasma)  Pplat-group  SI-group  p-value | 341 (159 - 2512)  474 (198 - 1058)  1,00 | 1045 (198 - 4347)  1009 (579 - 3902)  1,00 |
| TNFalpha (BAL)  Pplat-group  SI-group  p-value | 977 (551 - 1073)  1033 (263 - 1061)  1,00 | 1070 (1024 - 2644)  972 (574 - 2820)  0,42 |
| IL-8 (Plasma)  Pplat-group  SI-group  p-value | 0 (0 - 7)  0 (0 - 0)  1,00 | 226 (29 - 392)  16 (0 - 230)  0,42 |
| IL-8 (BAL)  Pplat-group  SI-group  p-value | 8 (25 - 1330)  30 (0 - 595)  0,54 | 3516 (1157 - 3708)  624 (509 - 2554)  0,09 |

Table 1. Time-points T_0_: Baseline measurement, 10 minutes after protocolized baseline ventilatory parameters, before lung injury. T_4_: 240 min after protocolized ventilatory parameters (open lung PEEP and protocolized VT corresponding to the study group) were adjusted. Data are presented as median (IQR). TNFalpha: Tumor necrosis factor (pg/ml), IL-8: Interleukin 8 (pg/ml). BAL: broncho-alveolar lavage. * When significant difference (p < 0,05) between Pplat-group vs SI-group.

Table 2. Histophatologic analysis

| SI-group | | | | Pplat-group | | | |
| --- | --- | --- | --- | --- | --- | --- | --- |
| Animal | Slide | Ventral lung | Dorsal lung | Animal | Slide | Ventral lung | Dorsal lung |
| 1 | 1 | 1 | 5 | 1 | 1 | 1 | 3 |
|  | 2 | 2 | 5 |  | 2 | 1 | 4 |
|  | 3 | 2 | 5 |  | 3 | 1 | 4 |
| 2 | 1 | 1 | 3 | 2 | 1 | 1 | 4 |
|  | 2 | 1 | 4 |  | 2 | 1 | 3 |
|  | 3 | 1 | 3 |  | 3 | 1 | 3 |
| 3 | 1 | 1 | 3 | 3 | 1 | 3 | 4 |
|  | 2 | 1 | 3 |  | 2 | 2 | 4 |
|  | 3 | 1 | 3 |  | 3 | 2 | 4 |
| 4 | 1 | 1 | 4 | 4 | 1 | 1 | 3 |
|  | 2 | 1 | 4 |  | 2 | 1 | 4 |
|  | 3 | 2 | 4 |  | 3 | 1 | 3 |
| 5 | 1 | 3 | 3 | 5 | 1 | 1 | 4 |
|  | 2 | 1 | 4 |  | 2 | 1 | 3 |
|  | 3 | 2 | 4 |  | 3 | 1 | 6 |
| 6 | 1 | 2 | 3 | 6 | 1 | 2 | 4 |
|  | 2 | 1 | 4 |  | 2 | 2 | 3 |
|  | 3 | 2 | 4 |  | 3 | 4 | 3 |
| 7 | 1 | 1 | 4 | 7 | 1 | 1 | 4 |
|  | 2 | 2 | 4 |  | 2 | 2 | 4 |
|  | 3 | 1 | 4 |  | 3 | 1 | 4 |
| 8 | 1 | 1 | 3 | 8 | 1 | 2 | 4 |
|  | 2 | 2 | 3 |  | 2 | 2 | 3 |
|  | 3 | 1 | 4 |  | 3 | 1 | 4 |
| Lung damage score [ | | 1(1-3) | 4(3-5) |  |  | 1(1-4) | 4(3-6) |

Table 2. Histopathologic analysis. The degree of lung damage was assessed using a modified VILI histological scoring system (31) according to the following four items: 1) alveolar congestion, 2) hemorrhage, and 3) infiltration or aggregation of neutrophils in airspace or the vessel wall, and 4) thickness of the alveolar wall/hyaline membrane formation. Each item was graded according to a five-point scale: 0 = minimal damage, 1 = mild damage, 2 = moderate damage; 3 = severe damage; and 4 = maximal damage. Lung damage score is presented as median (range).
